# Supplementary material for: Structure of the 30S translation initiation complex coupled to paused RNA polymerase and its potential for riboregulation
Source: Nat Commun. 2025 Dec 13;17:693. doi: 10.1038/s41467-025-67330-2 (PMC12820379; doi:10.1038/s41467-025-67330-2)
Supplement: Supplementary file 1 — Supplementary Information [file 41467_2025_67330_MOESM1_ESM.pdf]

Supplementary Information

Structure of the 30S translation initiation complex coupled to paused RNA polymerase and its potential for riboregulation

Johann J. Roske[1], Giulia Paris[1], Akanksha Goyal[2], Marina Rodnina[2], Nikolay Zenkin[3], Katarzyna J. Bandyra[1+*], Ben F. Luisi[1*]

[1]Department of Biochemistry, Sanger Building, University of Cambridge, Tennis Court Road, Cambridge CB2 1GA, UK
[2]Max Planck Institute for Multidisciplinary Sciences, Göttingen, Germany
[3]Centre for Bacterial Cell Biology, Biosciences Institute, Faculty of Medical Sciences, Newcastle University, Baddiley-Clark Building, Richardson Road, Newcastle Upon Tyne, NE2 4AX, UK
[+]Current address: Faculty of Chemistry, Biological and Chemical Research Centre, University of Warsaw, Zwirki i Wigury 101, 02-089, Warsaw, Poland
*Correspondence to: bfl20@cam.ac.uk and k.bandyra@uw.edu.pl

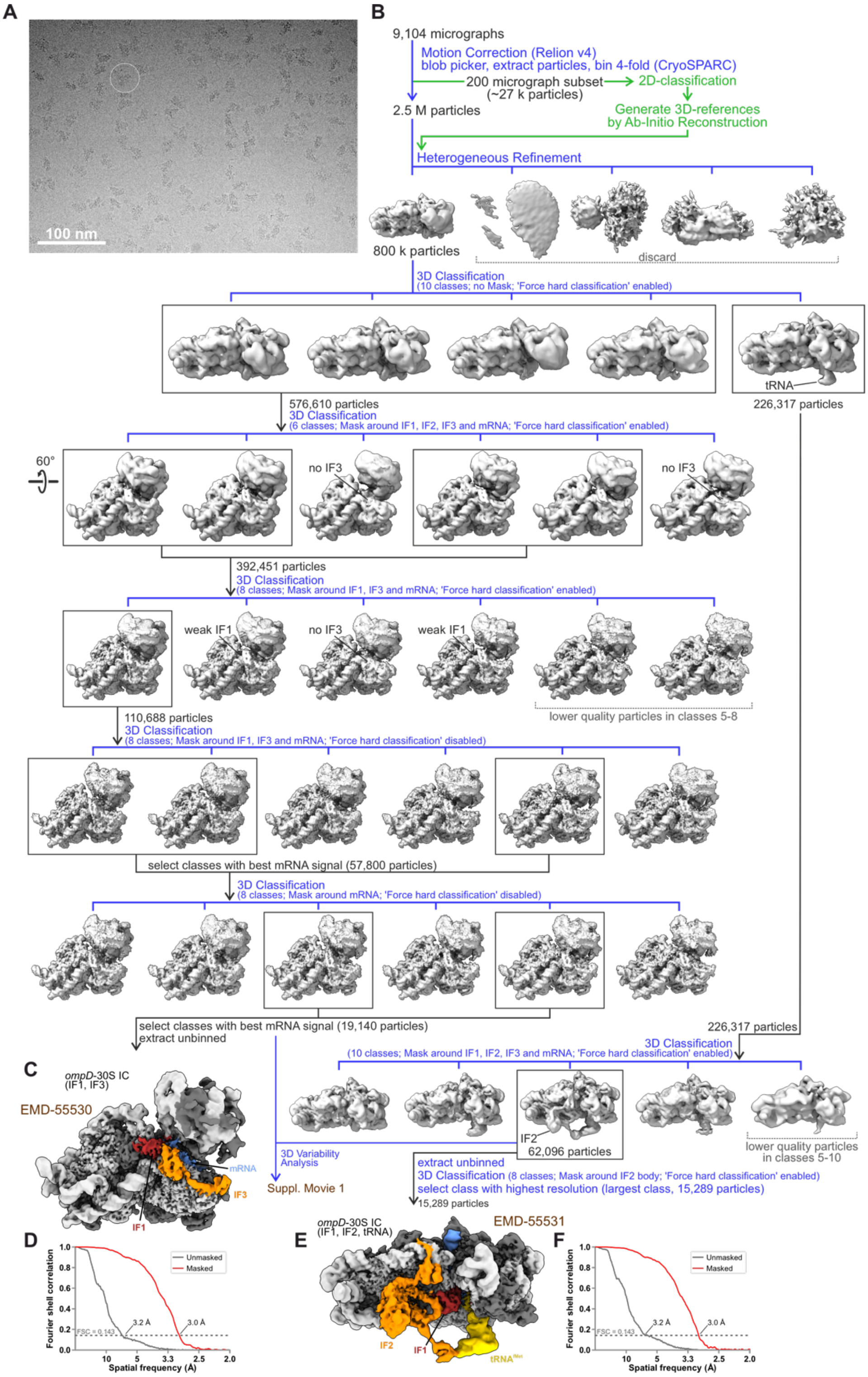

Supplementary Figure 1. Cryo-EM workflow for the 30S-*ompD* initiation complex.
(A) Representative electron micrograph. The white circle indicates a single 30S particle.
(B) Image processing workflow. Raw movies were motion-corrected in Relion v4 and micrographs were imported and further processed in CryoSPARC v4.
(C) Refined and locally filtered 3D map of the *ompD*-30S IC containing IF1 and IF3.
(D) Masked and unmasked Fourier Shell Correlation (FSC) plots for the final refinement of the *ompD*-30S IC containing IF1 and IF3. Global resolution is indicated at the FSC=0.143 cut-off.
(E) Refined and locally filtered 3D map of the *ompD*-30S IC containing IF1, IF2 and initiator tRNA.
(F) Masked and unmasked FSC plots for the final refinement of the *ompD*-30S IC containing IF1, IF2 and initiator tRNA. Global resolution is indicated at the FSC=0.143 cut-off.

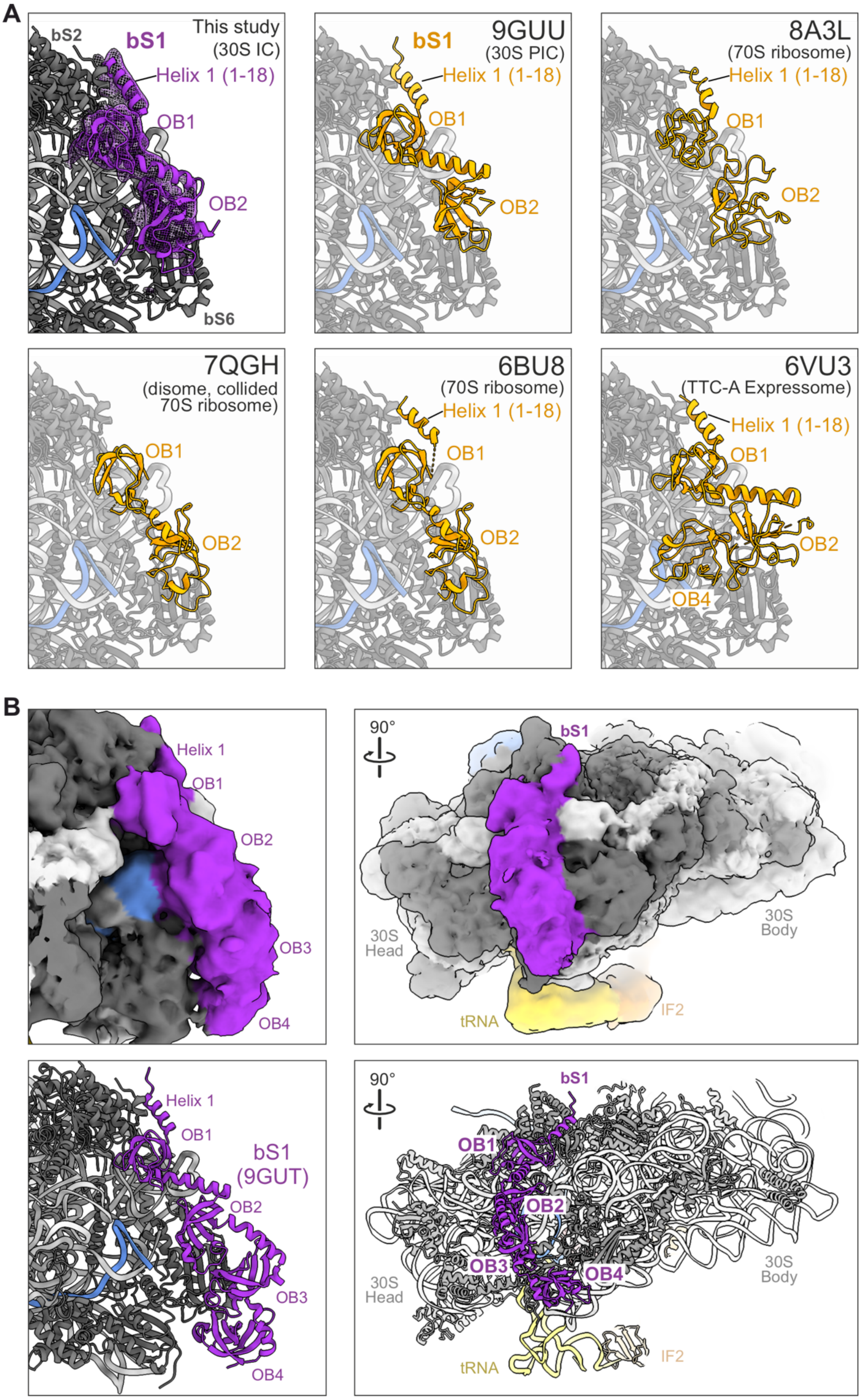

Supplementary Figure 2. Conformation of the bS1 protein.

(A) The conformation of the bS1 and its OB subdomains in this study (molecular model in purple ribbons, cryo-EM map is shown as mesh around bS1), and in earlier reports (yellow ribbon models). The mRNA is indicated as blue ribbon, 16s rRNA as grey ribbon, ribosomal proteins in dark grey ribbons.

(B) At lower thresholds, the cryo-EM map (colour-coded for bS1, purple, and mRNA, blue) shows additional density for the ensuing OB subdomains of bS1.

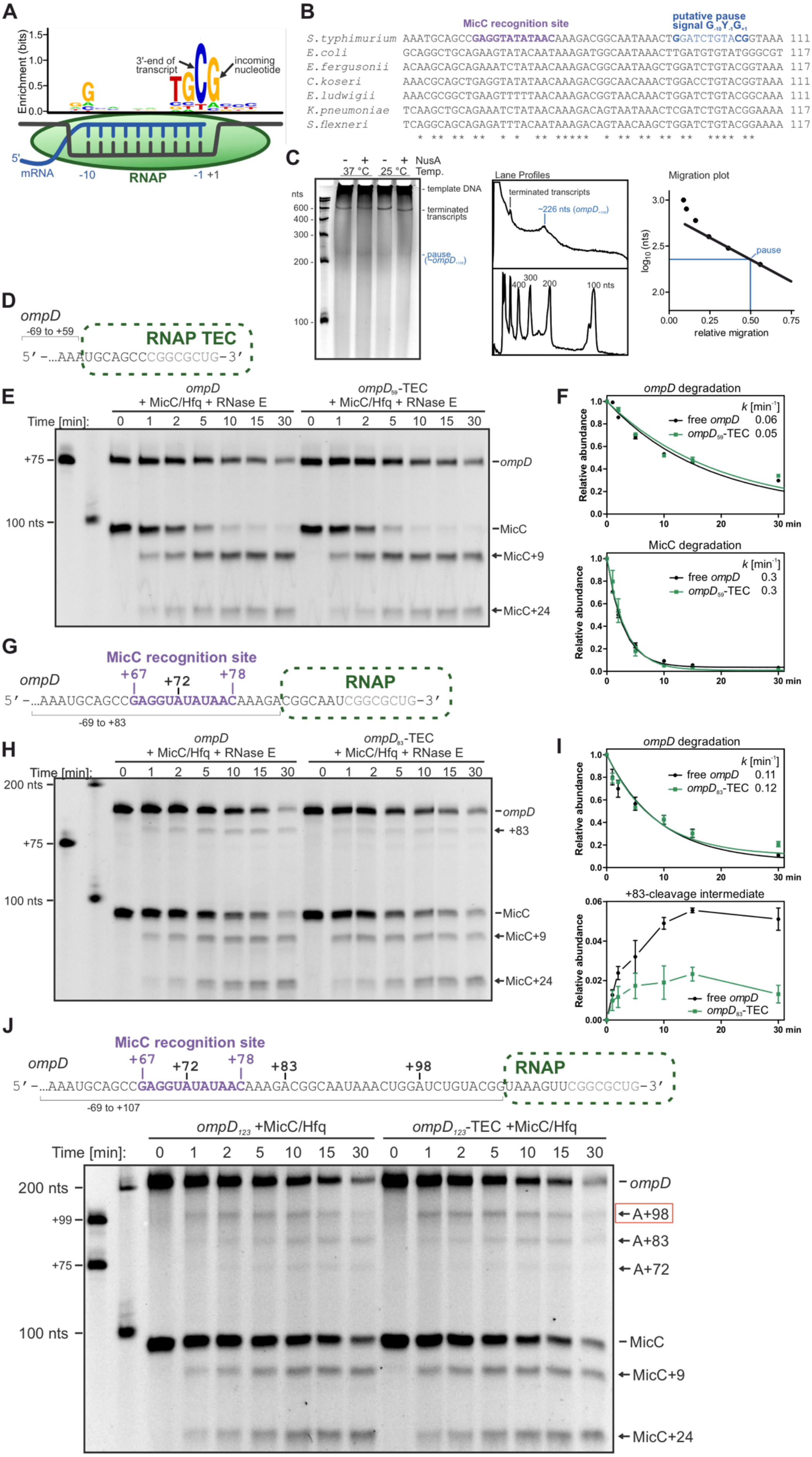
A
Enrichment (bits)
3'-end of transcript
incoming nucleotide
5'
mRNA
-10
RNAP
-1 +1
B
MicC recognition site
putative pause signal
S.typhimurium AAATGCAGCCGAGGTATATAACAAAGACGGCAATAAACTGGATCTGTACGGTAAA 111
E.coli GCAGGCTGCAGAAGTATACAATAAAGATGGCAATAAACTTGATGTGTATGGGCGT 117
E.fergusonii ACAAGCAGCAGAAATCTATAACAAAGACGGCAATAAGCTGGATCTGTACGGTAAA 117
C.koseri AAACGCAGCTGAGGTATATAACAAAGACGGCAACAAACTGGACCTGTACGGTAAA 111
E.ludwigii AAACGCGGCTGAAGTTTTTAACAAAGACGGTAACAAACTGGATCTGTACGGCAAA 111
K.pneumoniae TCAAGCTGCAGAAATCTATAACAAAGACAGTAATAAACTCGATCTGTACGGAAAA 117
S.flexneri TCAGGCAGCAGAGATTTACAATAAAGACAGTAACAAGCTGGATCTGTACGGAAAA 117
C
NusA
Temp.
37 °C
25 °C
template DNA
terminated transcripts
pause
Lane Profiles
~226 nts
Migration plot
log10 (nts)
relative migration
D
ompD
-69 to +59
RNAP TEC
5′-...AAAUGCAGCCCGGCGCUG-3′
E
ompD + MicC/Hfq + RNase E
ompD59-TEC + MicC/Hfq + RNase E
Time [min]: 0 1 2 5 10 15 30 0 1 2 5 10 15 30
+75
100 nts
ompD
MicC
MicC+9
MicC+24
F
ompD degradation
k [min-1]
free ompD 0.06
ompD59-TEC 0.05
Relative abundance
MicC degradation
free ompD 0.3
ompD59-TEC 0.3
G
MicC recognition site
ompD
+67 +72 +78
RNAP
5′-...AAAUGCAGCCGAGGUAUAUAACAAAGACGGCAAUCGGCGCUG-3′
-69 to +83
H
ompD + MicC/Hfq + RNase E
ompD83-TEC + MicC/Hfq + RNase E
Time [min]: 0 1 2 5 10 15 30 0 1 2 5 10 15 30
200 nts
+75
100 nts
ompD
+83
MicC
MicC+9
MicC+24
I
ompD degradation
k [min-1]
free ompD 0.11
ompD83-TEC 0.12
+83-cleavage intermediate
free ompD
ompD83-TEC
J
MicC recognition site
ompD
+67 +72 +78 +83 +98
RNAP
5′-...AAAUGCAGCCGAGGUAUAUAACAAAGACGGCAAUAAACUGGAUCUGUACGGUAAAGUUCGGCGCUG-3′
-69 to +107
ompD123 +MicC/Hfq
ompD123-TEC +MicC/Hfq
Time [min]: 0 1 2 5 10 15 30 0 1 2 5 10 15 30
200 nts
+99
+75
100 nts
ompD
A+98
A+83
A+72
MicC
MicC+9
MicC+24

Supplementary Figure 3. Degradation reactions of reconstituted *ompD*-TECs.

(A) HMMER logo for the transcription pause sequence adapted from (Imashimizu et al., 2015). Bases indicate sequences of the non-template DNA strand. The TEC is sketched with melted DNA in grey, nascent mRNA in blue and RNAP in green. In the $G_{-10}Y_{-1}G_{+1}$ pause motif, position -1 corresponds to the 3'-end of the nascent mRNA, with Y representing a pyrimidine base.

(B) Multiple sequence alignment of the *ompD* gene. Positions that code for the MicC recognition site, or mark a putative transcription pause site are highlighted. The putative pause site corresponds to the pause sequence in (A) in the positions $G_{-10}Y_{-1}G_{+1}$.

(C) *In vitro* transcription reactions in which *ompD* was transcribed by RNAP from the *ompD*-pMMB67HE plasmid at indicated temperatures in the absence or presence of NusA. Reactions were quenched by EDTA/SDS and analysed by SYBR gold-stained denaturing PAGE. PAGE lane profiles are shown in the middle panel for averaged signal of *in vitro* transcription reactions (upper profile) and molecular marker bands (lower profile). The long product ('terminated transcripts') corresponds in length to transcription from the *tac* promoter until the T1 terminator in the pMMB67HE vector backbone, between which a 306 bp-segment of the *ompD* gene (positions -69 to +237) were inserted. The short transcript species was approximated to a length of 226±3 nts by interpolation between 200 nts and 400 nts standards after plotting logarithmic molecular marker sizes vs. migration distances. When accounting for vector-originated nucleotides and the length of the *ompD* 5'-UTR, the paused transcript species corresponds to transcription stalling at *ompD* position +109±3.

(D-F) Time-course degradation reactions of *ompD* and *ompD*-TEC by RNase E 1-598 in the presence of MicC/Hfq using an *ompD* fragment that exposes positions -69 to +59 of the nascent mRNA. Graphs display degradation rates of *ompD* and MicC in the absence (free *ompD*, black) or presence of TEC (green). Data are mean ± SD from three independent reactions.

(G-I) Time-course experiment of MicC/Hfq-guided degradation of *ompD* and *ompD*-TEC by RNase E using an *ompD* fragment that exposes positions -69 to +83 of the nascent mRNA. Graphs display the degradation rate of *ompD* and the relative abundance of the +83-cleavage intermediate in the absence (free *ompD*, black) or presence of TEC (green). Data are mean ± SD from three independent reactions.

(J) Time-course degradation reactions of *ompD* and *ompD*-TEC by RNase E in the presence of MicC/Hfq using an *ompD* fragment that exposes positions -69 to +107 of the nascent mRNA. Schematics in D, G, and J display sequences of the 3' segments of respective *ompD* mRNA constructs. Terminal sequence segments for bubble formation are shown in light grey letters. The recognition sequence for MicC (where applicable) is marked in purple. RNase E cleavage sites are annotated at +72, +83 and +98. Green ovals delineate RNA nucleotides that are buried within the RNA polymerase (RNAP) in reconstituted *ompD*-TECs.

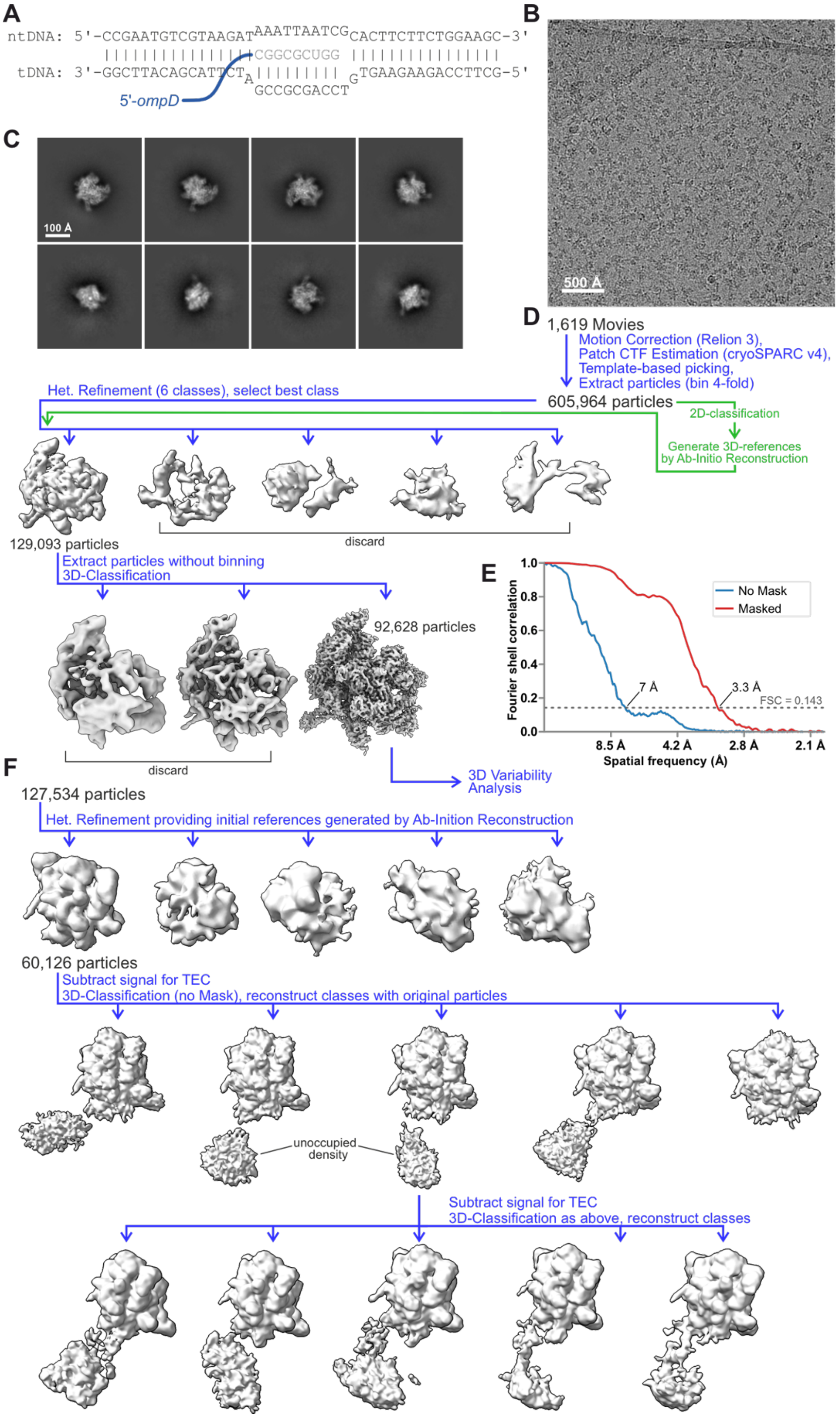

Supplementary Figure 4. Cryo-EM analysis of the *ompD*-TEC.
(A) Schematic of synthetic nucleic acid construct for TEC reconstitution.
(B) Representative cryo-electron micrograph. The scale bar indicates 50 nm.
(C) Representative 2D-class averages generated from the final particle set, the scale bar indicates 10 nm.
(D) Cryo-EM image processing workflow for *ompD*-TEC sample 1.
(E) Masked and unmasked FSC plots for the final Homogenous Refinement. Global resolution is indicated at the FSC=0.143 cut-off.
(F) Cryo-EM image processing workflow for *ompD*-TEC sample 2.

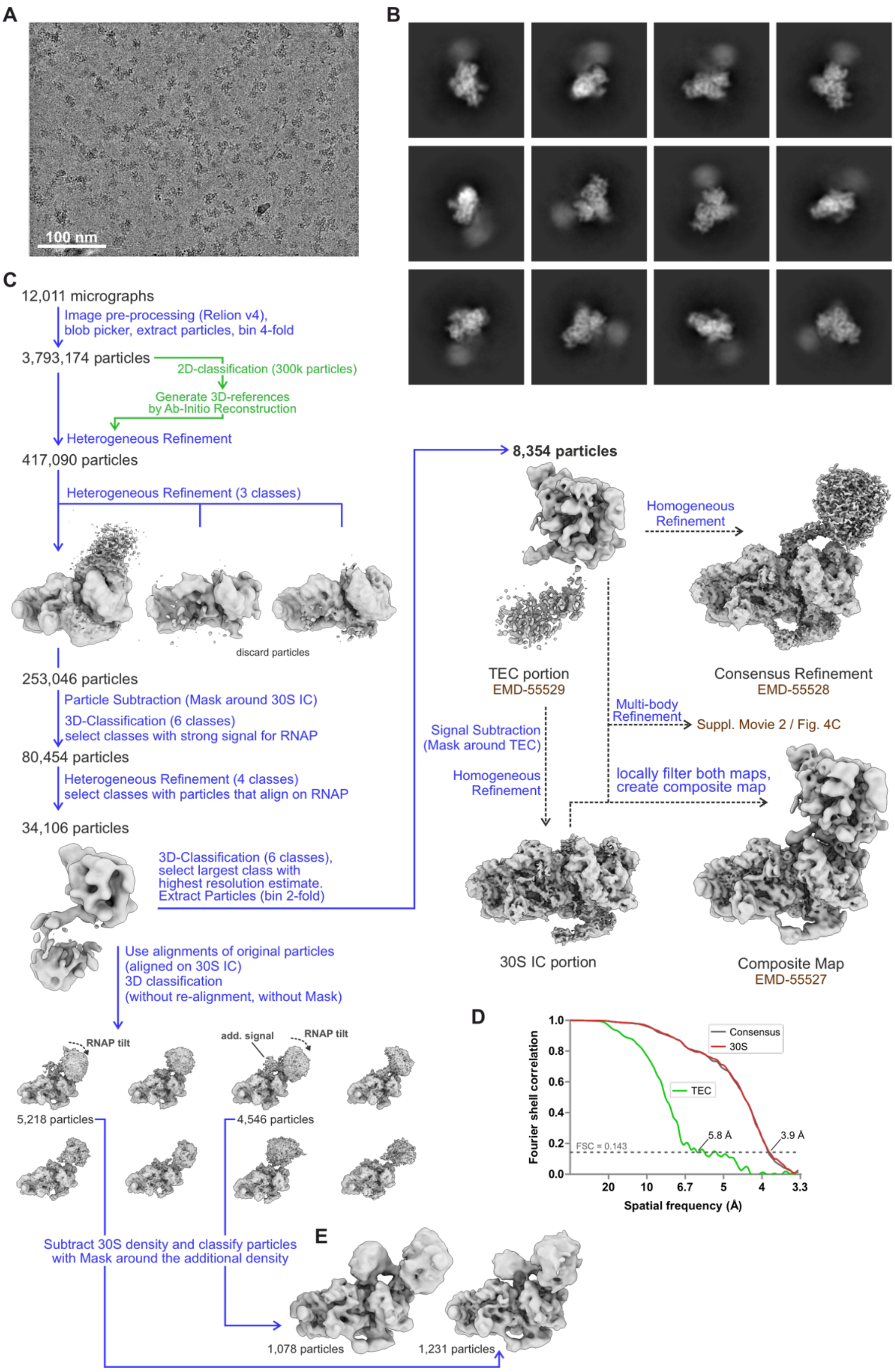

Supplementary Figure 5: Image processing overview for the coupled transcription elongation-translation initiation complex.
(A) Representative electron micrograph, est. defocus 2.0 μm, low pass filtered at 2 A.
(B) Representative 2D classes of the final particle set.
(C) Cryo-EM image processing workflow.
(D) Fourier Shell Correlation (FSC) plots for the three final refinements. Resolution is indicated at the FSC=0.143 cut-off.
(E) Cryo-EM Reconstructions of TEC-30S IC complexes with additional unoccupied density in the mRNA path between RNAP and 30S.

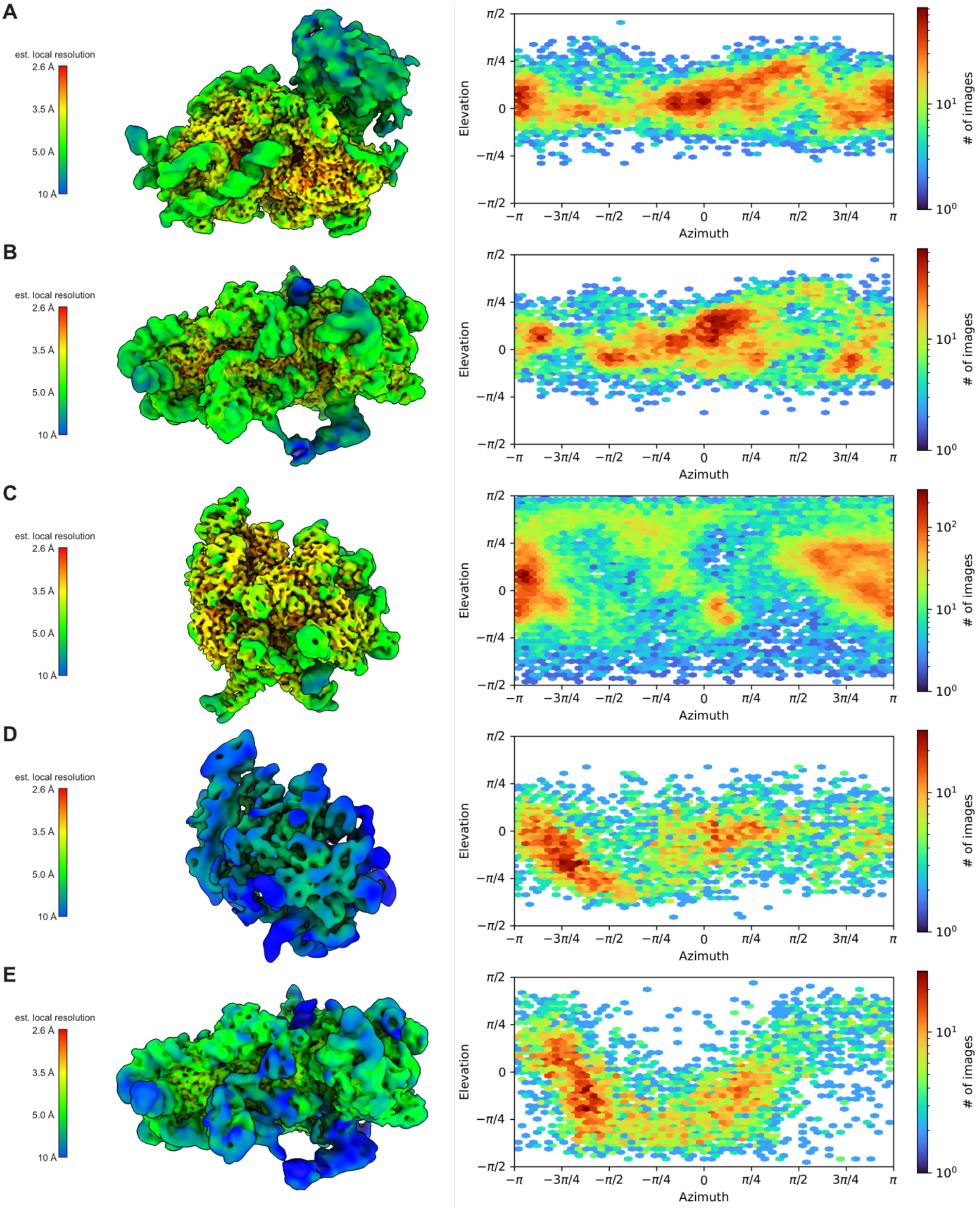

Supplementary Figure 6. Local resolution and particle orientation. Refined and locally filtered 3D maps are colored by local resolution as calculated in CryoSPARC. Particle orientation distribution plots of final refinements. A, *ompD*-30S IC-IF1-IF3; B, *ompD*-30S IC-IF1-IF2-tRNA; C, *ompD*-TEC; D, *ompD*-TEC portion of the TEC-30S IC complex; E, *ompD*-30S IC portion of the TEC-30S IC complex.

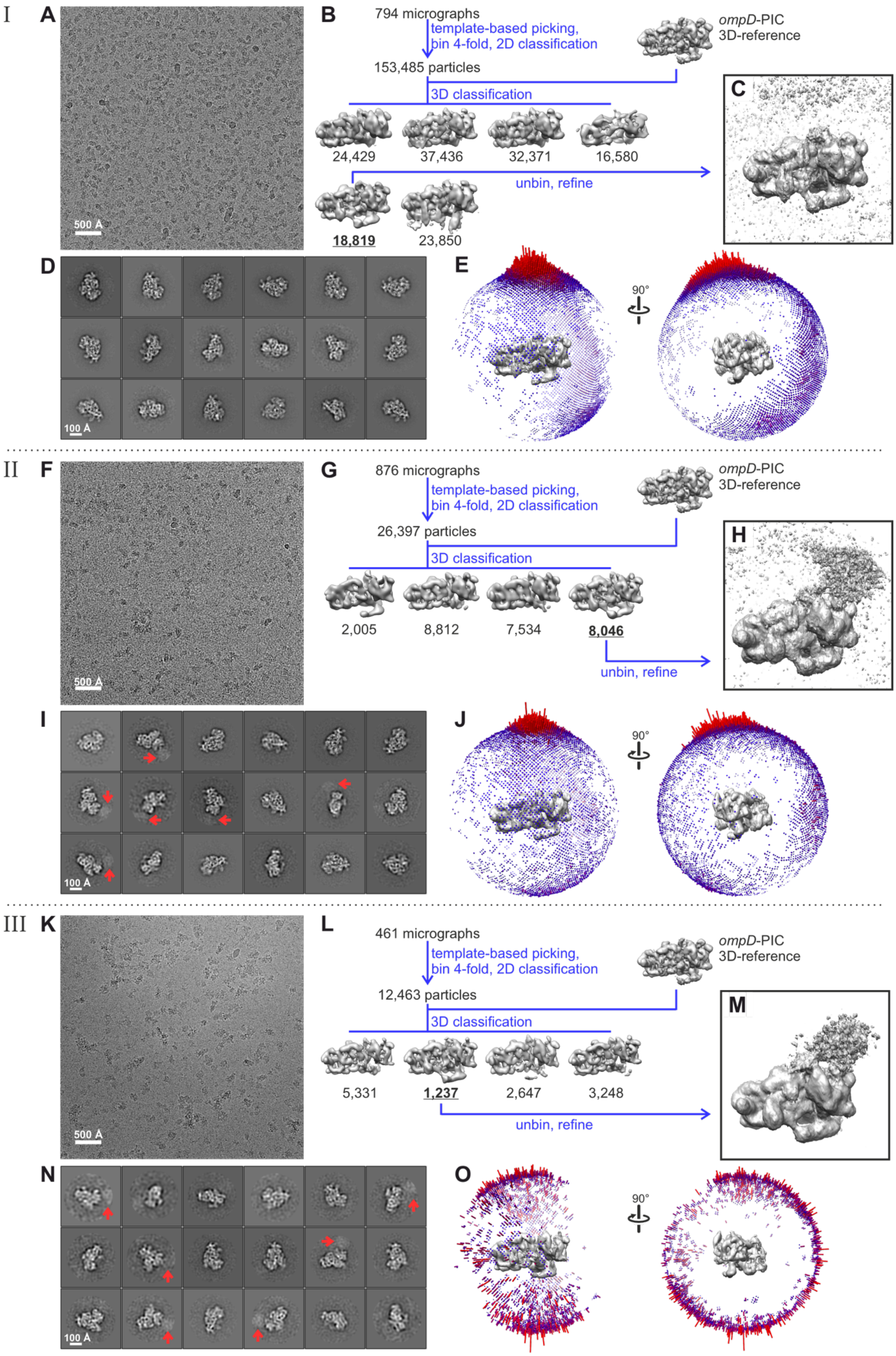
I
A
500 Å
B
794 micrographs
template-based picking,
bin 4-fold, 2D classification
153,485 particles
ompD-PIC
3D-reference
3D classification
24,429
37,436
32,371
16,580
unbin, refine
18,819
23,850
C
D
100 Å
E
90°
II
F
500 Å
G
876 micrographs
template-based picking,
bin 4-fold, 2D classification
26,397 particles
ompD-PIC
3D-reference
3D classification
2,005
8,812
7,534
8,046
unbin, refine
H
I
100 Å
J
90°
III
K
500 Å
L
461 micrographs
template-based picking,
bin 4-fold, 2D classification
12,463 particles
ompD-PIC
3D-reference
3D classification
5,331
1,237
2,647
3,248
unbin, refine
M
N
100 Å
O
90°

Supplementary Figure 7. Structural characterization of transcription-translation-coupled complexes. Three different samples were prepared for cryo-EM single particle analysis. (A-E) Sample I contains *ompD*-TEC and 30S IC in the absence of Nus-factors, vitrified on 2 nm amorphous carbon support. (F-J) Sample II was prepared as Sample I but in the presence of Nus-factors NusA and NusG. (K-O) Sample III was also prepared in the presence of Nus-factors and was additionally treated with glutaraldehyde prior to vitrification on graphene oxide-coated grids. (A, F, K) Representative micrographs, recorded with a 200 kV Talos Arctica, scale bars indicate 50 nm. (B, G, L) Image processing flow charts. (C, H, M) Final reconstructions shown at low contouring level. (D, I, N) Representative 2D classes from classification of particles from final structures. Red arrows indicate signal for RNAP. Scale bars are 10 nm. (E, J, O) Spherical representation plot of angular distributions of particle projections. Red bars indicate higher, blue bars lower abundance.
Cryo-EM data was collected on a 200 kV FEI Talos Arctica equipped with a Falcon 3 detector in linear mode. Images were recorded at 92,000x fold magnification at a pixel size of 1.13 Å with dose rates between 0.58 and 0.63 e$^-$/pixel/s and across a defocus range of -3.0 to -1.0 µm.

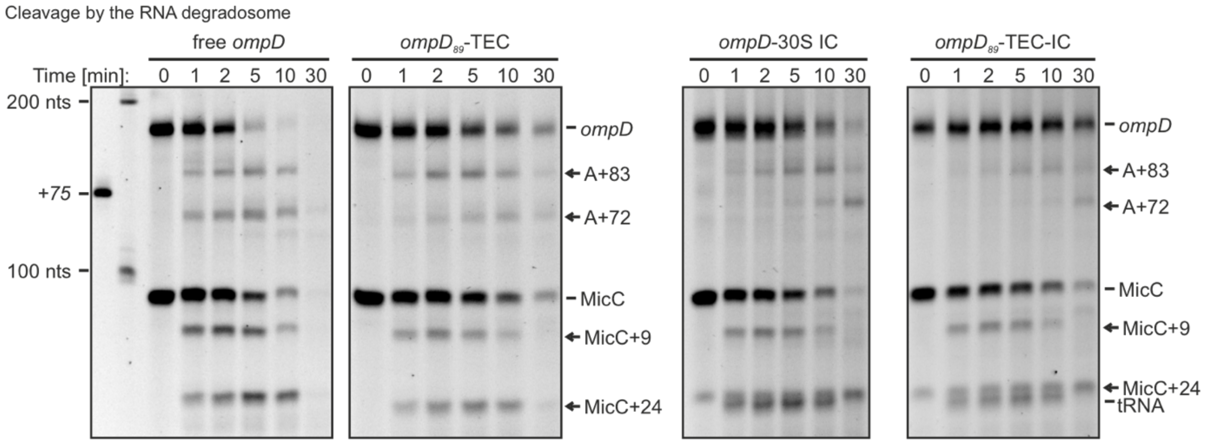

Supplementary Figure 8. Raw gels of degradation assays quantified in Figure 4. Representative time course reactions of *ompD* mRNA cleavage by the RNA degradosome in the presence of Hfq and MicC. Shown are reactions with free *ompD* mRNA fragment, *ompD*$_{89}$-TEC, *ompD*$_{89}$-30S IC and *ompD*$_{89}$-TEC-IC. Reactions were stopped at indicated time points and analysed by denaturing PAGE.

Supplementary Tables

Supplementary Table 1. Statistics of cryo-EM data collection, processing and refinement.

	ompD-30S IC		*ompD*-TEC	*ompD*-TEC-30S IC	
**Data collection and processing**					
Microscope	FEI Titan Krios		FEI Titan Krios	FEI Titan Krios	
Voltage (kV)	300		300	300	
Camera	Gatan K3		Falcon 3	Gatan K3	
Magnification	105,000		96,000	105,000	
Pixel size at detector (Å)	0.83		0.827	0.831	
Total electron dose ($e^-/Å^2$)	50		45.15	40.1	
Exposure rate (e^-/px/s)	15.309		0.67	16.296	
Frames per exposure	42		40	40	
Defocus range (μm)	-2.5 to -0.8		-2.4 to -0.6	-2.5 to -0.8	
Micrographs collected	9,104		1,619	12,011	
Total extracted particles	2,466,579		605,964	2,419,553	
**Reconstruction**	**State 1**	**State 4**	***ompD*-TEC**	**TEC**	**30S IC**
Final particle (no.)	19,140	15,289	41,790	8,354	8,354
Point-group symmetry	C1	C1	C1	C1	C1
Resolution (global, Å)	3.0	3.0	3.3	5.8	3.9
