# Supplementary material for: Structure of the 30S translation initiation complex coupled to paused RNA polymerase and its potential for riboregulation
Source: Nat Commun. 2025 Dec 13;17:693. doi: 10.1038/s41467-025-67330-2 (PMC12820379; doi:10.1038/s41467-025-67330-2)
Supplement: Supplementary file 2 — Description of Additional Supplementary Files [file 41467_2025_67330_MOESM2_ESM.pdf]

Description of Additional Supplementary Files

Supplementary Movie 1: 3D variability analysis of the 30S IC. 3D variability analysis indicates that the head region of the 30S subunit has a lower degree of mobility with respect to the 30S body, compared to the structure that contains IF3 but lacks fMet-tRNA^{fMet}

Supplementary Movie 2: Multi-Body Refinement for the TEC 30S IC. When the cryo-EM particles of the final subset are aligned on either 30S IC or RNAP, the respective other component remains only visible as noisy density (**Figure 3B; Suppl. Figure 5**). Multi-body Refinement in RELION allows the analysis of the flexibility between TEC and 30S [48]. The first three eigenvectors account for 50.8 % of the variance in the data (19.8, 16.1 and 14.9 % for components 1, 2 and 3, respectively) which is visualised **Figure 3C** and **Suppl. Movie 2**.
